# Cytotoxic Effect of Progesterone, Tamoxifen and Their Combination in Experimental Cell Models of Human Adrenocortical Cancer

**DOI:** 10.3389/fendo.2021.669426

**Published:** 2021-04-26

**Authors:** Elisa Rossini, Mariangela Tamburello, Andrea Abate, Silvia Beretta, Martina Fragni, Manuela Cominelli, Deborah Cosentini, Constanze Hantel, Federica Bono, Salvatore Grisanti, Pietro Luigi Poliani, Guido A. M. Tiberio, Maurizio Memo, Sandra Sigala, Alfredo Berruti

**Affiliations:** ^1^ Department of Molecular and Translational Medicine, Section of Pharmacology, University of Brescia at ASST Spedali Civili di Brescia, Brescia, Italy; ^2^ Pathology Unit, Department of Molecular and Translational Medicine, University of Brescia at ASST Spedali Civili di Brescia, Brescia, Italy; ^3^ Medical Oncology Unit, Department of Medical and Surgical Specialties, Radiological Sciences, and Public Health, University of Brescia at ASST Spedali Civili di Brescia, Brescia, Italy; ^4^ Department of Endocrinology, Diabetology and Clinical Nutrition, University Hospital Zurich (USZ) and University of Zurich (UZH), Zurich, Switzerland; ^5^ Medizinische Klinik und Poliklinik III, University Hospital Carl Gustav Carus Dresden, Dresden, Germany; ^6^ Surgical Clinic, Department of Clinical and Experimental Sciences, University of Brescia at ASST Spedali Civili di Brescia, Brescia, Italy

**Keywords:** adrenocortical carcinoma, ACC cell lines, ACC primary cells, estrogen receptors, progesterone receptors, tamoxifen

## Abstract

Progesterone (Pg) and estrogen (E) receptors (PgRs and ERs) are expressed in normal and neoplastic adrenal cortex, but their role is not fully understood. In literature, Pg demonstrated cytotoxic activity on AdrenoCortical Carcinoma (ACC) cells, while tamoxifen is cytotoxic in NCI-H295R cells. Here, we demonstrated that in ACC cell models, ERs were expressed in NCI-H295R cells with a prevalence of ER-*β* over the ER-*α*.Metastasis-derived MUC-1 and ACC115m cells displayed a very weak ER-*α*/*β* signal, while PgR cells were expressed, although at low level. Accordingly, these latter were resistant to the SERM tamoxifen and scarcely sensitive to Pg, as we observed a lower potency compared to NCI-H295R cells in cytotoxicity (IC_50_: MUC-1 cells: 67.58 µM (95%CI: 63.22–73.04), ACC115m cells: 51.76 µM (95%CI: 46.45–57.67) and cell proliferation rate. Exposure of NCI-H295R cells to tamoxifen induced cytotoxicity (IC_50_: 5.43 µM (95%CI: 5.18–5.69 µM) mainly involving ER-*β*, as their nuclear localization increased after tamoxifen: Δ A.U. treated *vs* untreated: 12 h: +27.04% (p < 0.01); 24 h: +36.46% (p < 0.0001). This effect involved the SF-1 protein reduction: Pg: −36.34 ± 9.26%; tamoxifen: −46.25 ± 15.68% (p < 0.01). Finally, in a cohort of 36 ACC samples, immunohistochemistry showed undetectable/low level of ERs, while PgR demonstrated a higher expression. In conclusion, ACC experimental cell models expressed PgR and low levels of ER in line with data obtained in patient tissues, thus limiting the possibility of a clinical approach targeting ER. Interestingly, Pg exerted cytotoxicity also in metastatic ACC cells, although with low potency.

## Introduction

Adrenocortical carcinoma (ACC) is a rare and aggressive tumor with an incidence of 0.7–2 new cases per million populations per year (1). Early diagnosis followed by radical surgical resection associated or not with adjuvant mitotane therapy (2, 3) is the only option that can give to ACC patients a chance of cure (4). The standard systemic treatment for advanced/metastatic ACC patients, not eligible to surgery, is mitotane, which is administered either alone or in combination with Etoposide, Doxorubicin, and Cisplatin (EDP-M regimen) (5). Although some pathological responses have been observed (6), the efficacy of EDP-M is limited and most initially responding patients are destined to relapse and die of the disease. Other cytotoxic therapies administered to patients with disease progression to EDP-M did not show a remarkable activity (7, 8). Molecular target therapies, attempted up to now (9), and immunotherapy (10) appeared ineffective.

Progesterone receptors (PgRs) and estrogen receptors (ERs) are expressed at different intensities in both normal and neoplastic adrenal cortex (11); however, the patho-physiological relevance of the steroid receptor expression in the physiological regulation of adrenal cell proliferation is not yet fully understood. In particular, in adulthood, ER-*β* is expressed in the glomerular and fasciculated area of the adrenal cortex, while at the prepubertal age, it is mainly located in the reticular area (12, 13). The ER-*α* subtype appears to be poorly expressed. During the course of neoplastic degeneration, there is an unpredictable rearrangement of the expression of these receptors, and data concerning the expression of the ERs are controversial. Indeed, a negativity for ER-*α* and an increase of the ER-*β* in the AdrenoCortical Carcinoma (ACC) have been reported by immunohistochemical analysis (11), while a decrease of ER expression has been observed as the ACC progresses (14, 15). Finally, other studies demonstrated low ER-*β* levels and/or high levels of ER-*α* in numerous cases of ACC, leading to an increase in the ER-*α*/ER-*β* ratio compared to that observed in healthy tissue (13). In the NCI-H295R cells, it was observed that ER-*β* gene expression is higher compared to ER-*α*, and the selective estrogen receptor modulator (SERM) 4-OH-tamoxifen inhibits cell proliferation (16).

The expression of ER subtypes varies in different tissues, although they are often co-expressed (17). The traditional paradigm is that ER-*α* is oncogenic and increases cell survival, while ER-*β* exerts an opposite role, being protective and pro-apoptotic. This clear distinction, however, cannot be applied for each tissue and cell expressing both ER subtypes; indeed, ER-*α* has a dominant role in tissues such as the uterus, mammary glands, pituitary, skeletal muscle, adipose, and bone; whereas, ER-*β* has a major role in the ovary, prostate, lung, cardiovascular, and central nervous systems (17).

PgR expression was as well detected in ACC (11). Recently, our group demonstrated a cytotoxic effect of Pg in ACC cells (18). Pg treatment of NCI-H295R cells induced apoptosis *via* activation of PgR with the involvement of both genomic and non-genomic pathways.

In breast cancer cells, PgR is a transcriptional target of ER, and estrogen is well known to be an important stimulator of PgR synthesis (19). Similar results have been obtained in human endometrial carcinoma (20). Interestingly, in a rare and peculiar setting such as pregnancy in ACC patients, in which there are elevated levels of both Pg and E hormones, their role in the control/progression of the disease is controversial. Indeed a study in 12 pregnant ACC patients concluded that pregnancy is associated with shorter survival and disease-free survival compared to control group (21), while another study on 17 treated ACC patients becoming pregnant during the follow-up, the pregnancy seems to be not associated with worse clinical outcome (22). As the authors correctly pointed out, however, pregnancy-associated ACC tended to be discovered at a more advanced stage. Thus, the possibility of a pregnancy-induced more rapid progression cannot be excluded, and we would like to underline that diagnostic and therapeutic delays probably account for the most severe presentation. Tamoxifen and medroxy-progesterone acetate combined treatment exhibited significant inhibitory growth effect on breast cancer (23), endometrial cancer (24), and cisplatin-resistant ovarian cancer cells (25). This combination therapy appeared to be active in phase II studies enrolling endometrial carcinoma patients (26). These data provided the rationale to explore the cytotoxic interaction between selective estrogen receptor modulators (SERMs), such as tamoxifen, and Pg in ACC.

Here, we explored the possible effect of tamoxifen on ACC cell viability and investigated the additive/synergic cytotoxic activity of tamoxifen and progesterone in *in vitro* ACC experimental cell models.

## Materials and Methods

### Cell Lines

The human NCI-H295R cell line, derived from a primitive ACC in a female patient (27), was obtained from the American Type Culture Collection (ATCC) and cultured as indicated by ATCC. MUC-1 cell line, established form a neck metastasis of an EDP-M treated male patient, was kindly given by Dr. Hantel and cultured as suggested (28). Media and supplements were supplied by Sigma Aldrich Italia, (Milan, Italy).

### Primary ACC Cell Culture

Human ACC primary cells were derived from a male patient who underwent surgical removal of metastatic ACC, in progression after EDP-M. The local Ethical Committee approved the project and written informed consent was obtained from the patient. The primary culture ACC115m was obtained as previously described (29) and maintained in MUC-1 medium supplemented with L-Glutamine (2 mM) and amphotericin B (2.5 μg/ml). The clinical characteristics of the patient are reported in **Supplemental Table 1**. Cells were tested for mycoplasma and authenticated from BMR genomics (Padova, Italy).

### Immunohistochemistry

Tissue samples were obtained from formalin-fixed and paraffin embedded blocks from surgical samples. 2 μm thick sections were used for routine Hematoxylin and Eosin (H&E) staining and immunohistochemistry using the automatic stainer BenchMark ULTRA IHC/ISH System (Ventana). Diagnosis of cortical cell carcinoma was revised according to the most recent WHO criteria (30). The clinical characteristics of the patient are reported in **Supplemental Table 1**. The following primary antibodies were used: anti-PgR clone 1E2, anti-ER clone SP1. All the primary antibodies were from “ready to use” kits from Ventana. Antigen retrieval was performed by incubation for 64 min for PgR and ER at 95°C in Ultra Cell Conditioning Solution (Ultra CC1, Ventana). Signal was revealed using the ultraView Universal DAB Detection kit (Ventana) followed by diaminobenzydine as chromogen and Hematoxylin for nuclear counterstain. Digital images were acquired by an Olympus XC50 camera mounted on a BX51 microscope (Olympus, Tokyo, Japan) using CellF Imaging software (Soft Imaging System GmbH, Münster, Germany). Expression of PR and ER was semi-quantitatively scored on representative tumor areas based on both percentage [score ranges: 0 (0–5%), 1 (6–29%), 2 (30–69%), 3 (≥70%)] and intensity (score ranges: 0, no expression; 1, weak; 2, moderate; 3, high) of immunoreactive (IR) neoplastic cells.

### Immunofluorescence

Cells were grown onto 12  mm poly-L-lysine coated coverslips for 4 days and were then fixed with paraformaldehyde 4% (w/v) (Immunofix, Bio-Optica, Milan, Italy) for 15 min at 4°C and permeabilized with 20% MetOH and 0.1% Triton X-100 in PBS for 10 min. Non-specific binding was blocked by incubation in PBS containing 0.1% Triton X-100 and 0.2% of BSA for 45 min. Cells were incubated with anti-PgR (raised in rabbit, 1:800, Cell Signaling Technology, Denvers, MA, USA), anti-ER-*β* (raised in rabbit, 1:500, Abcam, Cambridge, United Kingdom) and anti-ER-*α* (raised in mouse, 1:500, Invitrogen, Carlsbad, CA, USA) primary antibodies o/n at 4°C. After extensive washes, the anti-rabbit Alexa Fluor 488 (green signal) and anti-mouse Alexa Fluor 555 (red signal) (Immunological Sciences, Rome, Italy) secondary antibodies, and Alexa Fluor 647 Phalloidin (Invitrogen) were applied for 1 h at rt. After rinsing in PBS, coverslips were mounted using DAPI-containing Vectashield mounting medium (Vector Laboratories, Burlingame, CA, USA).

Slides were observed by a LSM 880 Zeiss confocal laser microscope equipped with Plan-Apochromat 63×/1.4 numerical aperture oil objective or by a LSM 510 Zeiss confocal laser microscope (Carl Zeiss AG, Oberkochen, Germany) equipped with Plan-Apochromat 63×/1.4 numerical aperture oil objective. Images were then reconstructed using Zeiss ZEN 2.3 Imaging Software (Carl Zeiss). The specific mean fluorescence intensity of the pixels was quantified using ZEN Black software (Carl Zeiss) and/or ImageJ software (Nation Institute of Health. Bethesda, MD, USA). Several fields, randomly chosen, were acquired and analyzed for each experimental condition.

### Cell Treatments

Cells were treated with increasing concentrations of progesterone (0.1–160 µM; Merck Serono, Milan, Italy) and tamoxifen (0.1–20 µM; Selleckchem Chemicals-DBA Italia, Segrate, Milan, Italy); both drugs were solubilized in DMSO. Preliminary experiments of concentration–response curves were conducted in the ACC cell cultures in order to establish the optimal drug concentration range and length of treatment. All experiments were conducted in charcoal-dextran-treated serum (CTS).

### Measurement of Cell Viability and Proliferation

Cell viability was assessed by 3-(4,5-Dimethyl-2-thiazol)-2,5-diphenyl-2H-tetrazolium bromide (MTT) dye reduction assay as described in Fiorentini et al. (31). Briefly, untreated and drug-treated cells were incubated with MTT dye (at final concentration of 0.5 mg/ml) and solubilized with DMSO. Absorbance was determined at 540/620 nm by a spectrophotometer (GDV, Rome, Italy). Cell proliferation rate was evaluated with TC20 automated cell counter (Bio-Rad Laboratories, Segrate, Milan, Italy). Briefly, cells were grown in 24-well plates, dislodged by trypsinization and suspended in culture medium followed by trypan blue dilution (1:2). The parameter settings were established according to the manufacturer’s instructions. 10 µl of sample was loaded into a slide and counted.

### Drug Combination Experiments

Combination experiments were performed to evaluate the interaction of Pg and tamoxifen on cell viability according to the Chou and Talalay method (32). Cells were treated for 4 days using increasing concentrations of progesterone (7.4–84.3 µM), tamoxifen (0.8–13.5 µM), and mitotane (1.51–17.21 µM) as single drug and in combination, as recommended for the most efficient data analysis (33). The drug concentration curve for the combination has been designed for each ACC cell model based on the respective IC_50_ of each drug. Data were then converted to Fraction affected (Fa, range from 0 to 1 where Fa = 0 indicating 100% cell viability and Fa = 1 indicating 0% cell viability) and analyzed using the CompuSyn software (ComboSyninc. Paramus, NJ, USA) to calculate the Combination Index (CI). A CI value <1, = 1, and >1 indicates synergism, additive effect, and antagonism respectively.

### Quantitative RT-PCR

Gene expression was evaluated by q-RT-PCR (ViiA7, Applied Biosystems, Milan, Italy) using SYBR Green as fluorochrome as described elsewhere (34). Sequences of oligonucleotide primers were reported in **Supplemental Table 2**. Reactions were performed under the following conditions: 1 cycle at 95°C for 10 min, 40 cycles at 95°C for 15 s, 62°C for 1 min. Differences of the threshold cycle (Ct) values between the *β* actin housekeeping gene and the gene of interest (ΔCt) were then calculated.

### miRNA Analysis

Total RNA, including miRNAs, was extracted from cells using the miRNeasy kit (Qiagen, Milan, Italy), and 1 µg was transcribed into cDNA using miScript II RT kit (Qiagen), following the manufacturer’s protocol. q-RT-PCR was performed with a miScript System (Qiagen) (35). Reactions were performed under the following conditions: 95°C 15 min; 94°C 15 s, 55°C 30 s, 70°C 30 s, 40 cycles. Sequences of miR-23 used were: miR23a: 5′AUCACAUUGCCAGGGAUUUCC; miRNA23b: 5′AUCACAUUGCCAGGGAUUACC. Variations in expression of miR-23a/b among different samples were calculated after normalization to U6.

### Western Blot

Cells were homogenized in cold RIPA buffer, and total protein concentrations were determined by Bio-Rad Protein Assay (Bio-Rad Laboratories). Proteins (30 μg/lane) were separated by electrophoresis on a 4–12% NuPAGEbis-tris gel system (Life Technologies, Milan, Italy) and electroblotted to a nitrocellulose membrane. Membranes were incubated with an anti-SF1 (0.234 µg/ml; Cell Signaling Technology) and anti-GAPDH (1 µg/ml Merk Millipore, Burlington, MA, USA) primary antibodies according to the manufacturer’s instructions. Secondary HRP-labeled anti-mouse and anti-rabbit antibodies (Santa Cruz Biotechnologies, Heidelberg, Germany) were used, and the specific signal was visualized using a Westar ECL Sun Western blot substrate (Cyanagen, Bologna, Italy). Densitometric analysis of the immunoblots was performed using NIH ImageJ Software.

### Statistical Analysis

The analysis of the data was carried out by the GraphPad Prism version 5.02 software (GraphPad Software, La Jolla, CA) using the one-way ANOVA with Bonferroni’s multiple comparisons test considering P < 0.05 as threshold for significant difference. IC_50_ values for each drug were calculated by non-linear regression of the concentration–response curves. All results are expressed as mean ± SEM of three independent experiments, unless otherwise specified. Cytotoxicity experiments were carried out at least three times, each point run in triplicate.

## Results

### Estrogens in the ACC Cell Models

**Figure 1:**
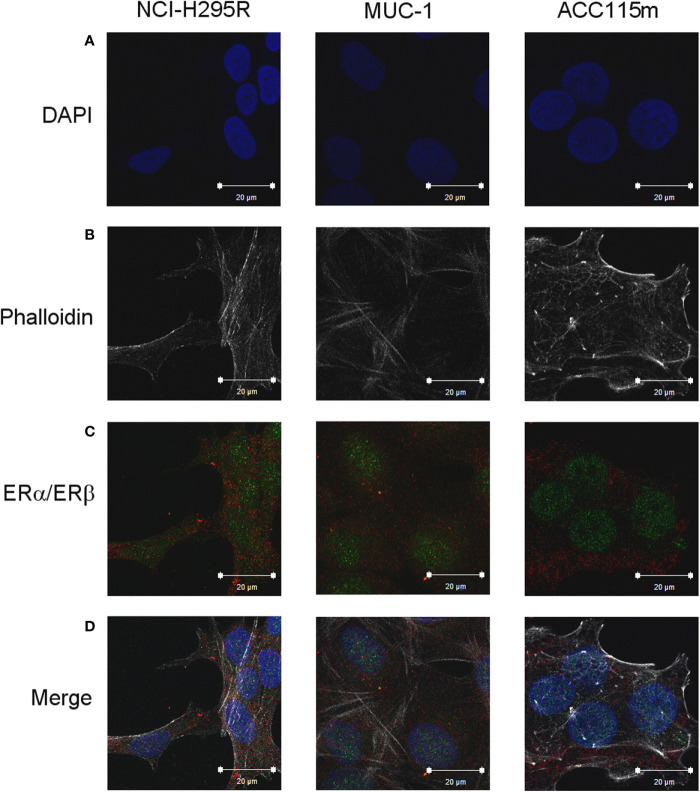
ER expression in NCI-H295R, MUC-1 cell lines and ACC115m primary culture. Cells were seeded on poly-L-lysine pre-treated coverslips following by incubation with DAPI for nuclear staining. Panel **(A)** DAPI; panel **(B)** phalloidin; panel **(C)** ER (red signal: ER-*α*; green signal: ER-*β*); panel **(D)** merge. The scale bar of 20 µm is automatically inserted by the software ZEN Black.

Due to the suggested different roles of ER in cell viability, we evaluated whether the ER-*α* and ER-*β* subtypes were differentially expressed in ACC experimental cell models. ACC cell lines and the ACC115m primary cell culture were then investigated for ER gene and protein subtype expression. Results on gene expression are reported in **Table 1**, while the mRNA translation into the respective protein was demonstrated by immunofluorescence and reported in **Figure 1** and quantified in **Supplemental Figure 1**. Concerning the ACC cell lines, NCI-H295R cells expressed both ER subtypes, although the gene and the protein both indicated a low level of expression with a prevalence of ER-*β* over the ER-*α* (**Figure 1A**, **Supplemental Figures 1**, **2**). Metastasis-derived MUC-1 cell line and ACC115m primary culture, displayed a very weak expression of ER-*α* and ER-*β*, both at gene (**Table 1**) and protein levels (**Figure 1**; quantified in the **Supplemental Figure 1**). We would like to underline the peculiar sub-cellular localization of the ER subtypes as we can observe a prevalent nuclear localization of ER-*β*.

**Table 1:** ER gene expression in ACC cell lines and primary cell culture.

Target gene	NCI-H295R	MUC-1	ACC115m
ER-*α*	10.88 ± 0.36	>15.00	11.50 ± 0.83
ER-*β*	9.81 ± 0.38	>15.00	13.43 ± 0.68

Values were reported as ΔCt that are differences of the threshold cycle (Ct) values between the β-actin housekeeping gene and the gene of interest (ΔCt), calculated, as described in Materials and Methods.

**Figure 2:**
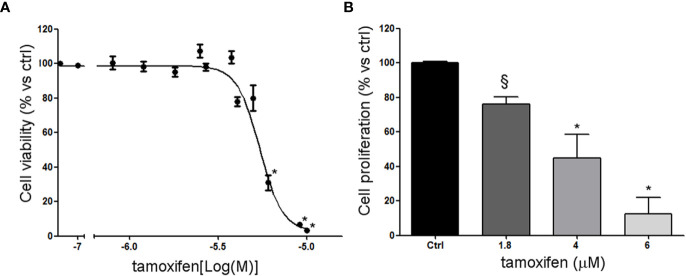
Effect of tamoxifen on NCI-H295R cell viability and proliferation. **(A)** NCI-H295R were treated with increasing concentration of tamoxifen (0.1–20 uM) and cell viability was then evaluated by MTT assay. Results are expressed as percent of viable cells *vs* ctrl ± SEM of three independent experiments run in triplicate. **(B)** NCI-H295R were treated with low, intermediate, and high dose of tamoxifen and then cell proliferation was evaluated by directing counting with trypan blue discrimination. *P < 0.0001 *vs* untreated cells; ^§^ *P* < 0.001 *vs* untreated cells.

NCI-H295R cell line expressed the CYP19A1 enzyme (31) and produced 17β-estradiol (10.01 ± 0.77 ng/ml; **Supplemental Methods**). As it has been shown that exogenous administration induced cell growth [16 and unpublished data], to explore the possible involvement of ERs in ACC cytotoxicity and cell proliferation rate, ACC cells were treated with increasing concentrations of tamoxifen for 4 days and then evaluated for cell viability. The ACC cell line NCI-H295R displayed a concentration-dependent cytotoxicity, with the IC_50_ of 5.43 µM (95% CI: 5.18–5.69 µM) (**Figure 2A**) and the reduction of the cell proliferation rate (**Figure 2B**). MUC-1 cell line and ACC115m primary culture resulted resistant to tamoxifen (**Supplemental Figure 3**), accordingly to the very low ER expression in these ACC cell models. In particular, tamoxifen exposure did not show any effect on cell viability up to 15 µM and then a sharp decrease at 17.5 µM and 20 µM, more evident in ACC115m. Whether this effect is ER-dependent or not needs to be determined.

### Tamoxifen Induced ER-β Nuclear Translocation in NCI-H295R Cell Line

**Figure 3:**
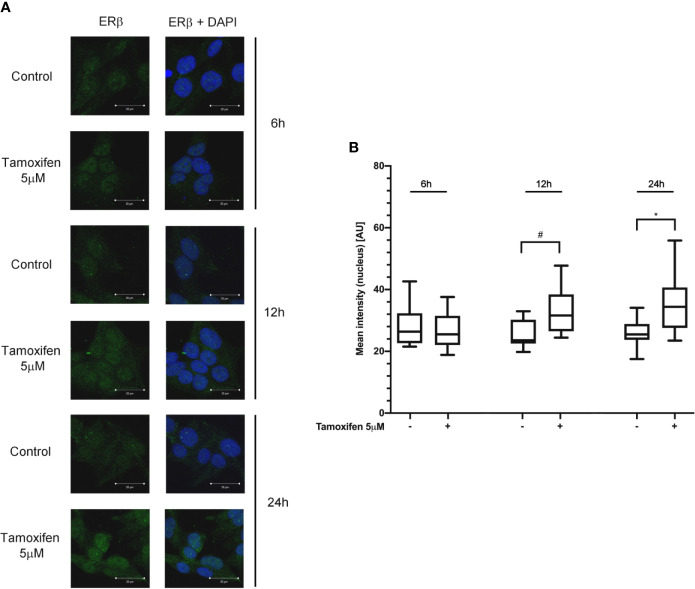
Tamoxifen exposure selectively modified the ER intracellular localization in NCI-H295R cells. **(A)** Cells were treated for different times with tamoxifen IC_50_ value. Slides were observed by a LSM 880 Zeiss confocal laser microscope or by a LSM 510 Zeiss confocal laser microscope (Carl Zeiss with 40× magnification. Images were then reconstructed using Zeiss ZEN 2.3 Imaging Software (Carl Zeiss). On the left the ER-*β* staining, on the right ER-*β* + DAPI staining. **(B)** The specific mean fluorescence intensity of the pixels of acquired images was quantified using ZEN Black software (Carl Zeiss). Several fields, randomly chosen, were acquired and then analyzed for each experimental condition. Quantified analysis was conducted by GraphPad Prism 5.02 software. ^*^P < 0.0001 *vs* ctrl; ^#^P < 0.01 *vs* ctrl.

To evaluate whether the tamoxifen effect involved a selective subtype, NCI-H295R cells were exposed to the drug IC_50_, and cells were fixed and analyzed at the confocal microscope at different times. **Figure 3A** shows that tamoxifen treatment induced a time-dependent increase of nuclear signal of ER-*β*, thus suggesting a significant nuclear translocation after 12 h of drug exposure that was maintained up to 24 h (**Figure 3B**), without any modification of the amount and localization of ER-*α* (**Supplemental Figure 4**). These results suggested that ER-*β* could be the subtype mainly involved in the tamoxifen effect.

### Pg in the ACC Cell Models

**Figure 4:**
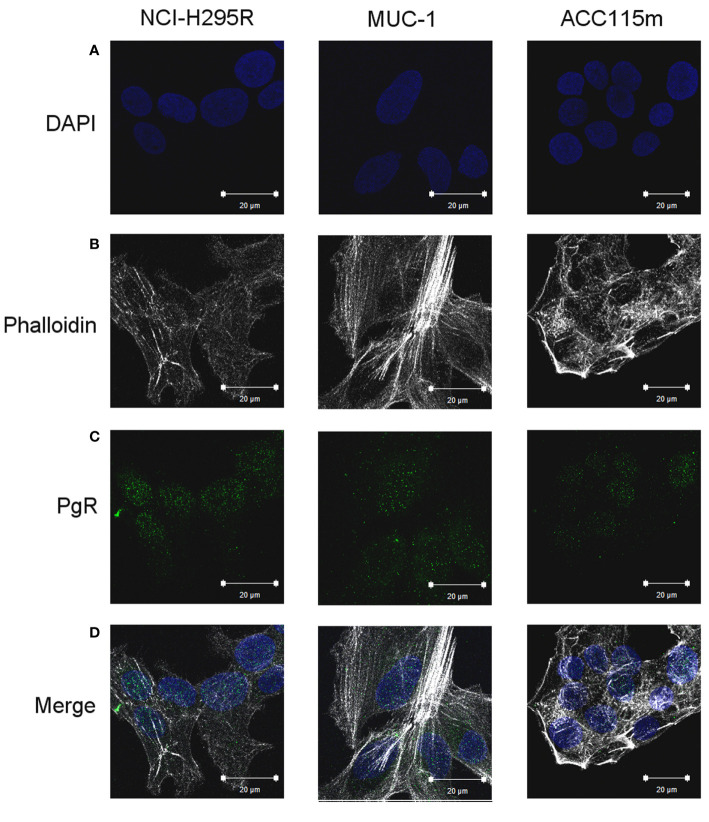
PgR expression in NCI-H295R, MUC-1 cell lines and ACC115m primary culture. Cells were seeded on poly-L-lysine pre-treated coverslips following by incubation with DAPI for nuclear staining. Panel **(A)** DAPI; panel **(B)** phalloidin; panel **(C)**: PgR; panel **(D)**: merge. The scale bar of 20 µm is automatically inserted by the software ZEN Black.

**Figure 5:**
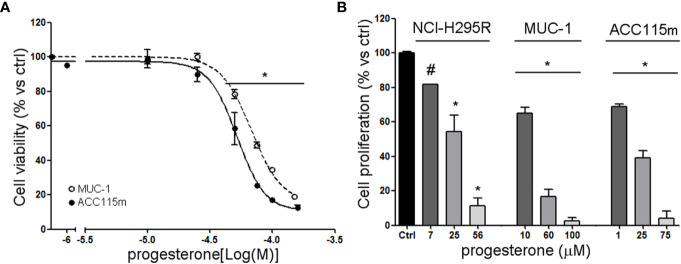
Cytotoxic effect of Pg in ACC cell models. **(A)** MUC-1 cell line and ACC115m primary culture were treated with increasing concentrations of progesterone (0.1–160 uM), then cell viability was analyzed by MTT assay, **(B)** NCI-H295R, MUC-1 cell lines and ACC115m primary culture were treated with low, intermediate, and high dose of Pg, and cell proliferation was analyzed by directing counting with trypan blue discrimination. Results are expressed as percent of viable cells *vs* ctrl ± SEM; *P < 0.0001 *vs* untreated cells; ^#^P < 0.01 *vs* untreated cells.

We already demonstrate that NCI-H295R cells express PgR (31) and that Pg exerts a concentration-dependent cytotoxic effect on NCI-H295R cells line as well as in ACC primary cell cultures expressing PgR (18). Here, we confirmed this result in other ACC cell models, studying the Pg effect in metastasis-derived cell models, namely MUC-1 cell line and in ACC115m primary cells. We firstly assessed the PgR expression in these cells by q-RT-PCR. The ΔCt obtained was MUC-1: 12.71 ± 0.62; ACC115m: 10.39 ± 0.04 (cDNA belonging from NCI-H295R cells was used as internal positive control: ΔCt: 9.48 ± 0.57), thus suggesting that PgR gene expression was present. Although a direct relationship between mRNA and proteins cannot be established, a correlation between the gene expression and the immunofluorescent signal in these ACC cell models could be observed. Indeed, PgR signal in MUC-1 cells and ACC115m primary cell culture is weaker compared to NCI-H295R cells. These results are reported in **Figure 4** and included NCI-H295R cells as positive control. The immunofluorescence signal quantification is reported in **Supplemental Figure 5**. A modest cytotoxic effect of both ACC cell models derived from metastatic patients was observed when cells were exposed to increasing Pg concentrations, suggesting that these cells were less sensitive to Pg compared to NCI-H295R cells. Indeed, the IC_50_ was 67.58 µM (95% CI: 63.22–73.04 µM) for MUC-1 cells and 51.76 µM (95% CI: 46.45–57.67 µM) for ACC115m cells (**Figure 5A**). Pg treatment affected as well the cell proliferation rate on each ACC cell model as reported in **Figure 5B**.

### Effect of Drug Combined Treatment on ACC Cell Viability

**Figure 6:**
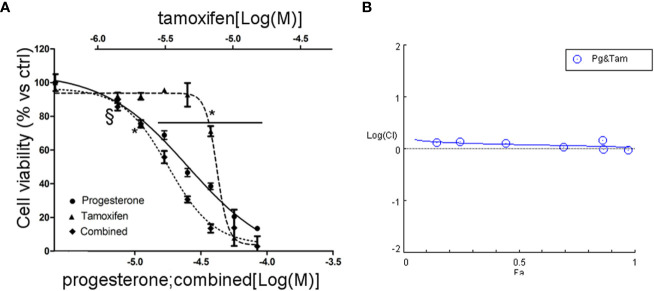
Combined treatment tamoxifen plus Pg in NCI-H295R. **(A)** Concentration–response curve of tamoxifen, Pg, and drug combination in NCI-H295R. Cells were exposed to increasing concentrations of tamoxifen and Pg alone or in combination as described in *Materials and Methods*. Data are expressed as percent of viable cells *vs* ctrl. Data are the mean ± SEM of three independent experiments; ^*^ *P* < 0.0001 *vs* untreated cells; ^§^ *P* < 0.001 *vs* untreated cells. **(B)** Combination index plot. Cell viability data of panel A were converted to Fa values and analyzed with CompuSyn software.

Due to the sensitivity of NCI-H295R cell line to both Pg and tamoxifen, we thus evaluated whether the cytotoxic effect of tamoxifen on NCI-H295R cell viability could be enhanced by Pg, applying the Chou–Talalay method for drug combination experiments (32, 33). Cells were exposed to increasing concentrations of tamoxifen (1.2–13.5 µM) and Pg (7.4–84.3 µM) at 1:6.17 fixed molar ratio for 4 days and then analyzed for cell viability by MTT assay (**Figure 6A**). The combination index was then calculated, and the analysis revealed a prevalent antagonist effect when the two drugs were combined (**Figure 6B**). The combination index value for each drug concentration is reported in **Supplemental Table 3**, and the isobolograms are reported in **Supplemental Figure 5**.

Finally, since mitotane is the standard treatment for ACC patients, we then evaluated as well the combined treatment NCI-H295R cell line with tamoxifen and mitotane. Results are reported in **Supplemental Figure 6**, **Supplemental Table 4** and showed that the combination has an additive/synergic effect at low concentrations, while, as the drug concentrations increased, the antagonism prevailed.

### Pg and Tamoxifen Reduced SF-1 Expression in NCI-H295R Cells

**Figure 7:**
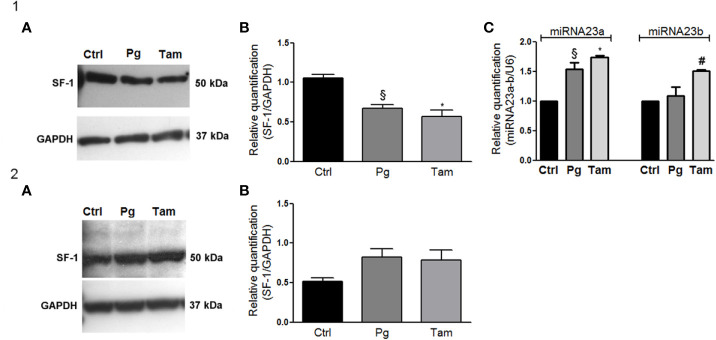
Tamoxifen and Pg reduced the SF-1 expression in NCI-H295R cell line. **(1A)** Representative western blot of SF-1 expression after NCI-H295R tamoxifen IC_50_ and Pg IC_50_ 4 days treatment. **(1B)** Densitometric analysis of SF-1 expression after NCI-H295R drug treatment. Data are expressed as normalized values SF-1/GAPDH and are the mean of three independent experiments. *P < 0.0001 *vs* ctrl; §P < 0.001 *vs* ctrl. **(1C)** NCI-H295R were treated with tamoxifen IC_50_ or Pg IC_50_ for 4 days and then miRNA23a/b expression was investigated. Data are expressed as normalized values on internal control U6 and are the mean of three independent experiments. ^*^P < 0.0001 *vs* ctrl; ^§^P < 0.001 *vs* ctrl; #P < 0.01 *vs* ctrl. **(2A)** Representative western blot of SF-1 expression after MUC-1 tamoxifen IC_50_ and Pg IC_50_ 5 days treatment. **(2B)** Densitometric analysis of SF-1 expression after MUC-1 drug treatment. Data are expressed as normalized values SF-1/GAPDH and are the mean of three independent experiments.

In order to evaluate the functional effect of Pg and tamoxifen in the NCI-H295R cell line, the effect of these drugs on the expression of the adrenal biomarker, namely SF-1, the pleiotropic transcription factor involved as well in the carcinogenesis (36) was studied. Cells were treated with Pg or tamoxifen at their respective IC_50_ for 4 days and then the SF-1 expression was evaluated. Results are reported in **Figure 7**. By q-RT-PCR, after Pg and tamoxifen treatment, no differences in the SF-1 gene expression were detected (not shown), while representative western blots were reported in **Figure 7.1A**. The SF-1 protein expression was modified by both drugs: in particular, as shown in **Figure 7.1B**, Pg treatment induced a significant SF-1 reduction in NCI-H295R cell line (Pg: −36.34% ± 9.26%; tamoxifen: −46.25% ± 15.68%; *P* < 0.01).

In order to explain this phenomenon, we investigated the expression of two miRNAs involved in SF-1 regulation, namely miR23a and miR23b (37). The reduction of SF-1 protein expression seemed to be mediated, at least in part, by the increase of miRNA 23a expression, with an increase compared to untreated cells of up to 1.54 ± 0.11 in Pg-treated cells and of 1.73 ± 0.04 in tamoxifen-treated cells respectively. An increase of miRNA-23b expression was as well observed after tamoxifen treatment (1.51 ± 0.02 compared to untreated cells), while this miRNA did not seem to be involved in the regulation of SF-1 protein expression when NCI-H295R cells are exposed to Pg (**Figure 7.1C**). SF-1 protein expression after Pg and/or tamoxifen IC_50_ treatment was measured also in MUC-1 cell line, but no significant variations were detected (**Figures 7.2A, B**).

### PgR and ER Expression in ACC Tissues

**Figure 8:**
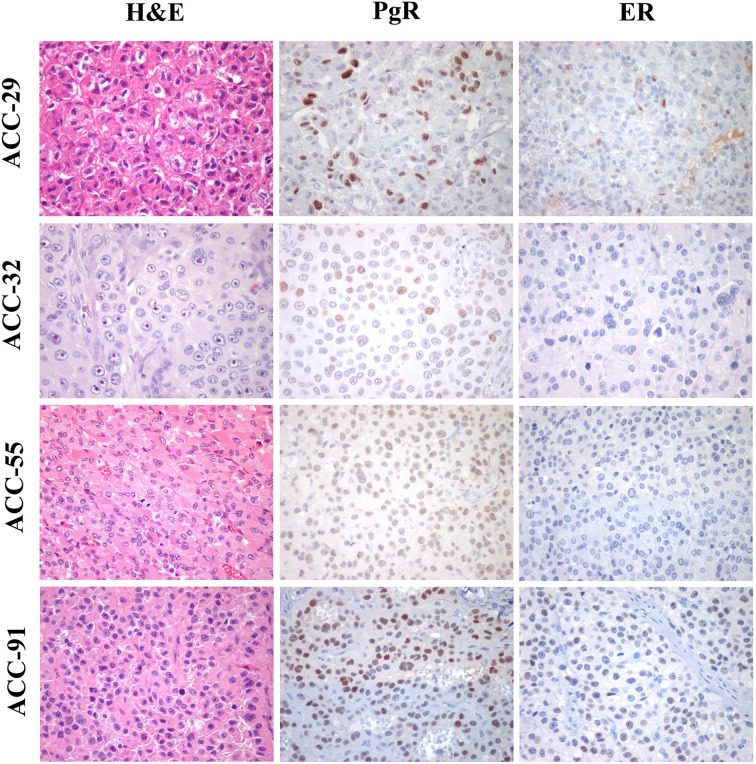
Immunohistochemistry for PgR and ER expression in ACC samples. Left panels show representative H&E-stained section from ACC tumor samples, middle panels PgR and right panels ER immunostainings. All images are from ×40 original magnification.

Finally, the expression of ER and PgR was studied by immunohistochemistry in 36 paraffin embedded tumor samples belonging to ACC diagnosed patients. Among this cohort, 13 patients were male and 22 female, with an age median of 53 years (range: 16–79 years), 11 of them were cortisol-secreting, while the others were not secreting. Results reported in **Table 2** indicated that ERs were absent or present in a very weak expression, while PgR proteins were expressed, although with a variability within the different samples. In particular, concerning the ER positive cells, we could observe that only three ACC samples displayed a percentage of ER moderately positive cells within the range of 30–69%, while 28 ACC displayed less than 5% ER positive cells, with a null o low intensity. Concerning PgR, they presented an evaluable expression in each sample studied, with only three ACC expressing less than 5% of immunoreactive cells. Indeed, almost half of samples expressed between 30 and 69% of immune positive cells and eight samples up to 36 expressed more than 70% of positive cells. A representative example of immunohistochemistry conducted on some ACC tissues is reported in **Figure 8**. The clinical characteristics are reported in **Supplemental Table 1**. In detail, ACC29 cells showed a tumor with lobulated morphology, moderate atypia and few mitotic figures. This tumor exhibits focal and moderate PgR expression, scant ER IR-cells, and low proliferation index. ACC32 cells presented an epithelioid morphology with higher nuclear atypia and prominent nucleoli. This tumor has few PgR IR cells with faint staining intensity with no ER expression and moderate proliferation index. ACC55 cells showed a solid growth composed of clusters of eosinophilic cells with frequent nuclear atypia and mitotic figures. Tumor has moderate PgR expression with negative ER immunostaining and a labeling index up to 15%. The ACC91 cells had a solid growth composed by poorly cohesive cell clusters with densely eosinophilic cytoplasm, frequent nuclear atypia and mitosis. This tumor has a higher expression of PgR along with moderate expression of ER. Labeling index is higher between these samples, ranging from 15 to 20%.

**Table 2:** Histological features and expression of PgR and ER in ACC tumor specimens.

code	PgR			ER		
	intensity	% of IR cells	cumulative	intensity	% of IR cells	cumulative
ACC03	1	2	**3**	0	0	**0**
ACC04	2	3	**5**	1	0	**1**
ACC06	1	1	**2**	2	2	**4**
ACC07	2	3	**5**	2	2	**4**
ACC08	1	2	**3**	0	0	**0**
ACC10	3	3	**6**	2	1	**3**
ACC11	2	2	**4**	0	0	**0**
ACC12	1	1	**2**	1	0	**1**
ACC13	1	2	**3**	0	0	**0**
ACC14	1	2	**3**	1	1	**2**
ACC16	2	2	**4**	1	0	**1**
ACC17	1	0	**1**	0	0	**0**
ACC23	1	0	**1**	0	0	**0**
ACC24	2	2	**4**	0	0	**0**
ACC26	1	2	**3**	0	0	**0**
ACC27	1	2	**3**	1	0	**1**
ACC29	2	1	**3**	1	0	**1**
ACC30	2	3	**5**	0	0	**0**
ACC32	1	1	**2**	0	0	**0**
ACC38	2	2	**4**	1	0	**1**
ACC40	1	1	**2**	0	0	**0**
ACC48	1	2	**3**	2	2	**4**
ACC50	1	2	**3**	1	0	**1**
ACC55	1	1	**2**	0	0	**0**
ACC64	2	3	**5**	0	0	**0**
ACC68	2	3	**5**	0	0	**0**
ACC71	2	2	**4**	1	2	**3**
ACC74	2	2	**4**	0	0	**0**
ACC75	1	1	**2**	0	0	**0**
ACC79	1	0	**1**	0	0	**0**
ACC81	2	2	**4**	0	0	**0**
ACC85	1	3	**4**	0	0	**0**
ACC91	2	3	**5**	1	2	**3**
ACC99	1	2	**3**	0	0	**0**
ACC103	1	2	**3**	0	0	**0**
ACC115	1	1	**2**	0	0	**0**

## Discussion

In this study, we *in vitro* investigated whether the interplay between ER, PgR, and their ligands may exert a cytotoxic and antiproliferative activity on ACC experimental cell models as it was demonstrated in endocrine-related cancers.

We observed that, although ER expression was relatively low, tamoxifen exerted cytotoxic effect on NCI-H295R cell line, belonging from a primitive ACC, confirming published data (16). Drug exposure led to an increased nuclear localization of ER-*β* subtype, with no modifications of the ER-*α* subcellular localization, leading to the hypothesis that the cytotoxic and antiproliferative effects of tamoxifen in ACC cells could be mediated by its ER-*β* agonist activity, according to previous observations (38). These results are in line with results showing that in breast cancer cell lines stably expressing ER-*β*, this receptor regulates multiple components normally associated with the suppression of cell proliferation (*i.e.* TGF*β* and cell cycle-related genes) (17). Thus, with these results, we supported evidence indicating that ER-*β* is a protective factor that suppresses uncontrolled proliferation and induces cell differentiation in many tissues and organs, both in physiological condition and in cancer degeneration (17). However, the role of ERs in ACC cell models seemed to be limited to the NCI-H295R cell line, as metastatic derived ACC cell models such as MUC-1 and ACC115m expressed very weak levels of both ER subtypes and were resistant to tamoxifen.

On the same line, this mechanism may have a scarce impact in clinic, as our immunostaining data showed that ER is scarcely expressed in paraffin-embedded ACC tissues as well as we observed in ACC experimental cell models, accordingly with those that detected low expression level of the ER subtypes in ACC.

The amount of ER expression in ACC seemed to decrease as disease progresses, at least in our experimental cell models. Indeed, as already underlined, in EDP-M resistant ACC cells, namely MUC-1 and ACC115m cells, the expression of ER is very low and cells do not respond to the SERM tamoxifen. Accordingly, in our cohort of paraffin-embedded ACC samples, the expression of ER was absent or present in a very weak expression, thus limiting the possibility to explore a clinical approach targeting ER in ACC patients. Another limitation resides in the tamoxifen pharmacokinetic, as the calculated plasma concentration at the steady state after 20 mg tamoxifen for 3 months is about 0.3 µM, that is under the range of concentrations that displayed a cytotoxic effect in our ACC experimental cell models, although tamoxifen presents a distribution volume that is about 50–60 l/kg (39).

Concerning PgR, immunohistochemical analysis of ACC tissues strongly indicated that they are frequently expressed, with a number of samples displaying a high percentage of immunoreactive cells, although with a large variability among samples. Accordingly, in a recent paper, our group demonstrated that exposure to Pg of primary cells derived from PgR expressing ACC (at least 40% of PgR+ cells) resulted in a concentration-dependent increase of cytotoxicity (18) in line with results demonstrating a role this hormone as anti-tumoral drug in different cancers (40–42).

Here, we strengthen the role of PgR in the ACC and the effect of Pg in reducing both cell proliferation and cell viability. This effect seemed to be strictly related to the level of PgR expression, thus the evaluation of the PgR expression during the pathological staging could be of interest, as Pg and its derivative are already part of the cancer supporting care, thus giving the opportunity to have another pharmacological tool over the usual systemic therapy. This hypothesis is now under study in a randomized phase II clinical trial.

The cross-talk between ER/PgR was detectable both at physiological and pathological levels in endocrine tissues and tumors (43). About it, it has been suggested that the combined treatment using drugs targeting ER/PgR could be useful, although the safety profile of the drug combination must be considered (43). Thus, as published data support the rationale for a synergism between anti-E and Pg in inducing an antineoplastic effect, we tested the cytotoxic activity of the combination of tamoxifen and Pg also in ACC experimental model of NCI-H295R cells. Results obtained indicated that the tamoxifen/Pg combination did not result in an either additive or synergic effect; rather the resulting effect was of drug antagonism.

We finally investigated the functional effect of tamoxifen and Pg exposure in ACC cell models, and we observed that both drugs are able to decrease the protein expression of the ACC biomarker SF-1, the transcription factor that is a critical regulator of adrenogonadal development and function (44). SF-1, also known as Ad4-binding protein or NR5A1, binds as a monomer to nuclear receptor half sites on DNA (44), and it plays an important role not only in adrenal steroidogenesis but also in cell adhesion, cell proliferation, apoptosis, and angiogenesis of adrenocortical tumor cells (36). Further, Doghman et al. demonstrated that overexpression of SF-1 in NCI-H295R increases proliferation rate (45). Thus, our results on the downregulation of SF-1 protein expression during the cytotoxic effect of tamoxifen and Pg on NCI-H295R cells found their rationale on the pleiotropic role of SF-1. The reduction of SF-1 protein expression along with a not significant modification of SF-1 mRNA expression induced by both drugs, led us to hypothesize that miRNA regulation of transcriptional capability of mRNA could occurred. It is indeed known that miRNAs, by binding to the 3′-untranslated region of target mRNAs, induced translational repression followed by degradation of approximately one-third of human genes (for an extensive review see: 46). Using computational approaches, it is suggested that each miRNA can bind to hundreds of different mRNAs, which collectively results in an extremely fine regulation of protein transcription (46). Thus, the concept that dysregulation of miRNA expression is linked to cancer is now accepted worldwide. Among the cancer-associated miRNAs, miR23a, one of the most studied miRNAs in different types of cancer, has been found to be involved, together with miR23b, in the regulation of SF-1 protein transcription (37). In NCI-H295R cells, we demonstrated that SF-1 reduction could be mediated, at least in part, by the increase of both miR23a and miR23b. The mechanism underlying this inverse correlation between SF-1 protein and miR23a and miR23b expression is still unknown; however, it has been shown that ER-*α* binding sites are present in the regulatory region of miR23a (47, 48) and miR23b, along with ER-*β* binding sites in miR23b regulatory region (49). To our knowledge, no evidence of a direct regulation of Pg on miR23a and miR23b is known at the moment; however, an indirect effect of Pg acting on E-ER-miR23a and miR23b regulation could be as well suggested, as it occurs for a large family of miRNAs in breast cancer (50).

Taken together, these results suggest that SF-1 expression seemed to be regulated by ER and PgR, These data, however, are not exhaustive and the full evidence of the inhibitory effect would require the demonstration of a modulation of the expression of other specific *β*-catenin target genes in NCI-H295R cells by Pg treatment. These further experiments are outside the scope of the present paper and will be a matter of a future study.

## Author’s Note

Part of these results has been presented at the 19th ENS@T Scientific Meeting—6 Nov 2020 ONLINE and accepted for presentation at the 40^th^National Meeting of the Italian Society of Pharmacology—9–13 March 2021 Digital Edition.

## Data Availability Statement

The original contributions presented in the study are included in the article/**Supplementary Material**. Further inquiries can be directed to the corresponding author.

## Ethics Statement

The studies involving human participants were reviewed and approved by Comitato Etico di Brescia. The patients/participants provided their written informed consent to participate in this study.

## Author Contributions

Conceptualization, SS, AB, and MF. Methodology, SS, ER, and MT. Formal analysis, AA, E.R, MF, and MC. Investigation, ER, MT, SB, AA, and MC. Writing—original draft preparation, ER, MT, and PP. Writing—review and editing, SS, AB, CH, PP, GT, and MM. Supervision, SS and AB. All authors contributed to the article and approved the submitted version.

## Funding

This work was funded by: AIRC project IG23009 (PI: AB), local grants from University of Brescia, Fondazione Camillo Golgi, Brescia and F.I.R.M. onlus Foundation, Cremona (Italy). CH received funding by the Uniscientia Foundation (keyword: tumor model).

## Conflict of Interest

The authors declare that the research was conducted in the absence of any commercial or financial relationships that could be construed as a potential conflict of interest.
