# Supplementary material for: Cytotoxic Effect of Progesterone, Tamoxifen and Their Combination in Experimental Cell Models of Human Adrenocortical Cancer
Source: Front Endocrinol (Lausanne). 2021 Apr 26;12:669426. doi: 10.3389/fendo.2021.669426 (PMC8108132; doi:10.3389/fendo.2021.669426)
Supplement: Supplementary file 1 [file DataSheet_1.docx]

**SUPPLEMENTARY DATA**

**1. Supplementary Methods**

*17β-Estradiol measurement using ELISA assay*

17β-Estradiol production was determined in the conditioned cell medium by an enzyme immunoassay method using Estradiol Human ELISA kit (Invitrogen, Carlsbad, CA, USA) according to the manufacturer’s instructions. Briefly, cells were plated at the density of 1 × 10^6^ cells/well in 6 wells plate in 1 ml of complete medium; 24 hours after seeded, supernatants were changed with charcoal-dextran-treated serum (CTS) medium. 24 hours later medium was collected and diluted 1:15 before testing. Medium alone was used as a blank. A standard 17β-Estradiol curve using a nonlinear regression analysis using the four-parameter logistic equation performed with GraphPad Prism version (5.02) was used to compare the absorbance values and to derive pg/mL 17β-estradiol concentration.

**2. Supplemental figures**

**Supplemental figure 1**

**
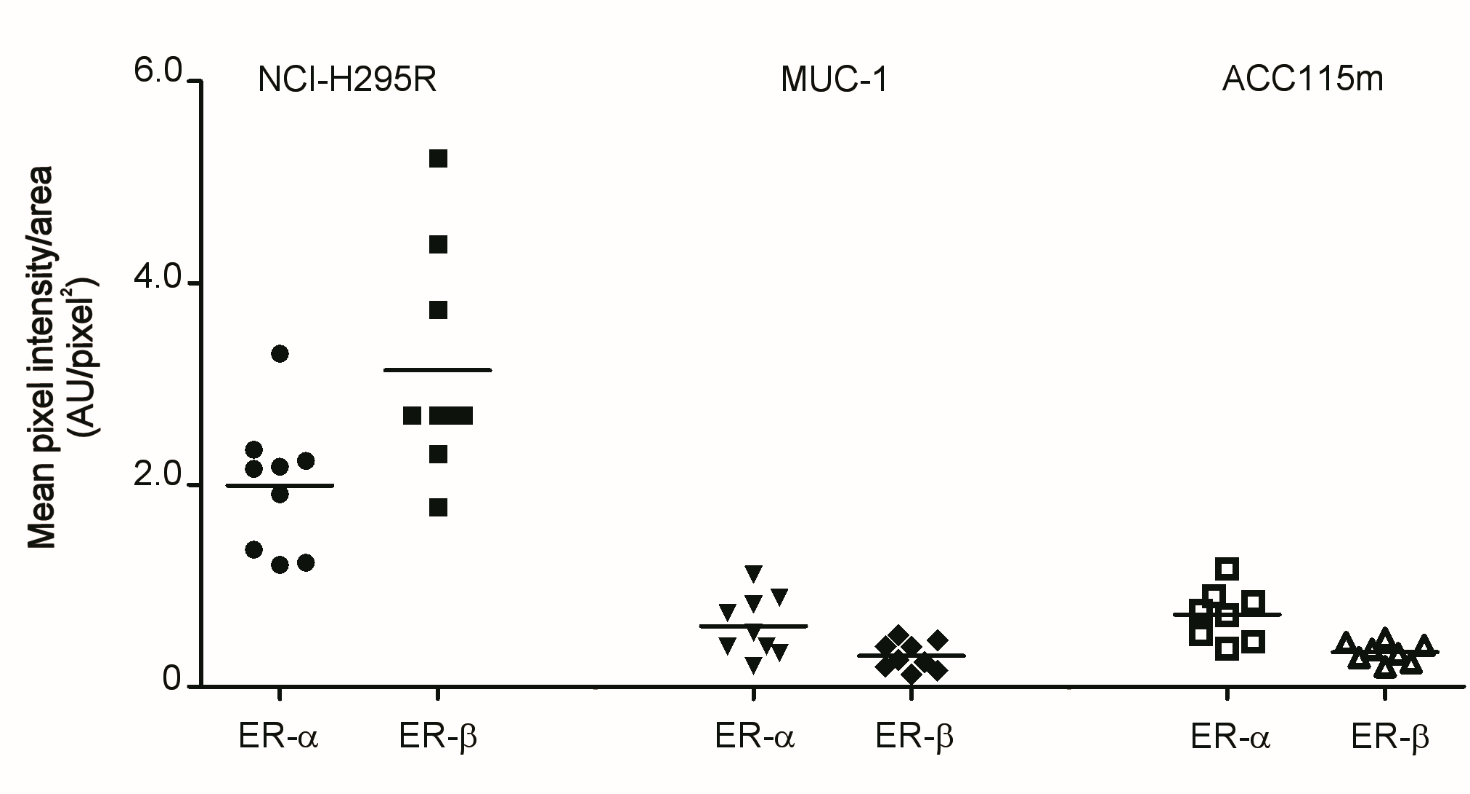
**

**Quantification of ER basal signal in ACC cell models.** The specific mean fluorescence intensity of the pixels of acquired images was quantified using NIH ImageJ software and normalized for area in order to minimize the differences dimension among each cell lines and primary culture. Several fields, randomly chosen, were acquired and then analyzed for each experimental condition. Quantified analysis was conducted by Graphad Prism 5.02 software.

**Supplemental figure 2**


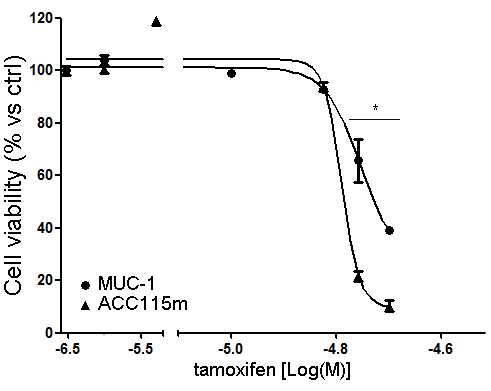


**Cytotoxic effect of tamoxifen on metastatic ACC cell models.** (A) Cells were treated with increasing concentrations of tamoxifen as describe in methods. Cell viability was evaluated by MTT assay. Results are expressed as percent of viable cells vs untreated cells. (B) Cells were treated with low, intermediate and high dose of tamoxifen and then the proliferation rate was estimated by directly counted as describe in methods. Data are the mean ± SEM of three independent experiments performed in triplicate. **P* < 0.0001.

**Supplemental figure 3**

**
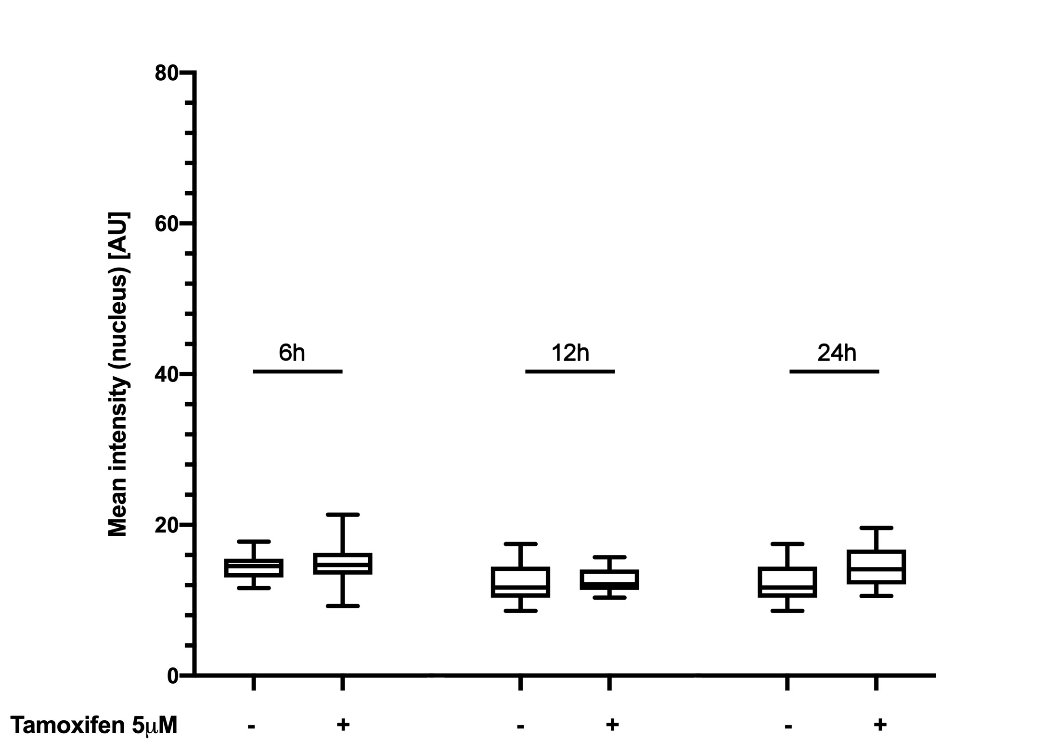
**

**Determination of ER-α translocation after tamoxifen treatment in NCI-H295R cell line.** Cells were treated with tamoxifen IC_50_ for different times as describe in methods. The specific mean fluorescence intensity of the pixels of acquired images was quantified using ZEN Black software (Carl Zeiss S.p.A). Several fields, randomly chosen, were acquired and then analyzed for each experimental condition. Quantified analysis was conducted by GraphPad Prism 5.02 software.

**Supplemental figure 4
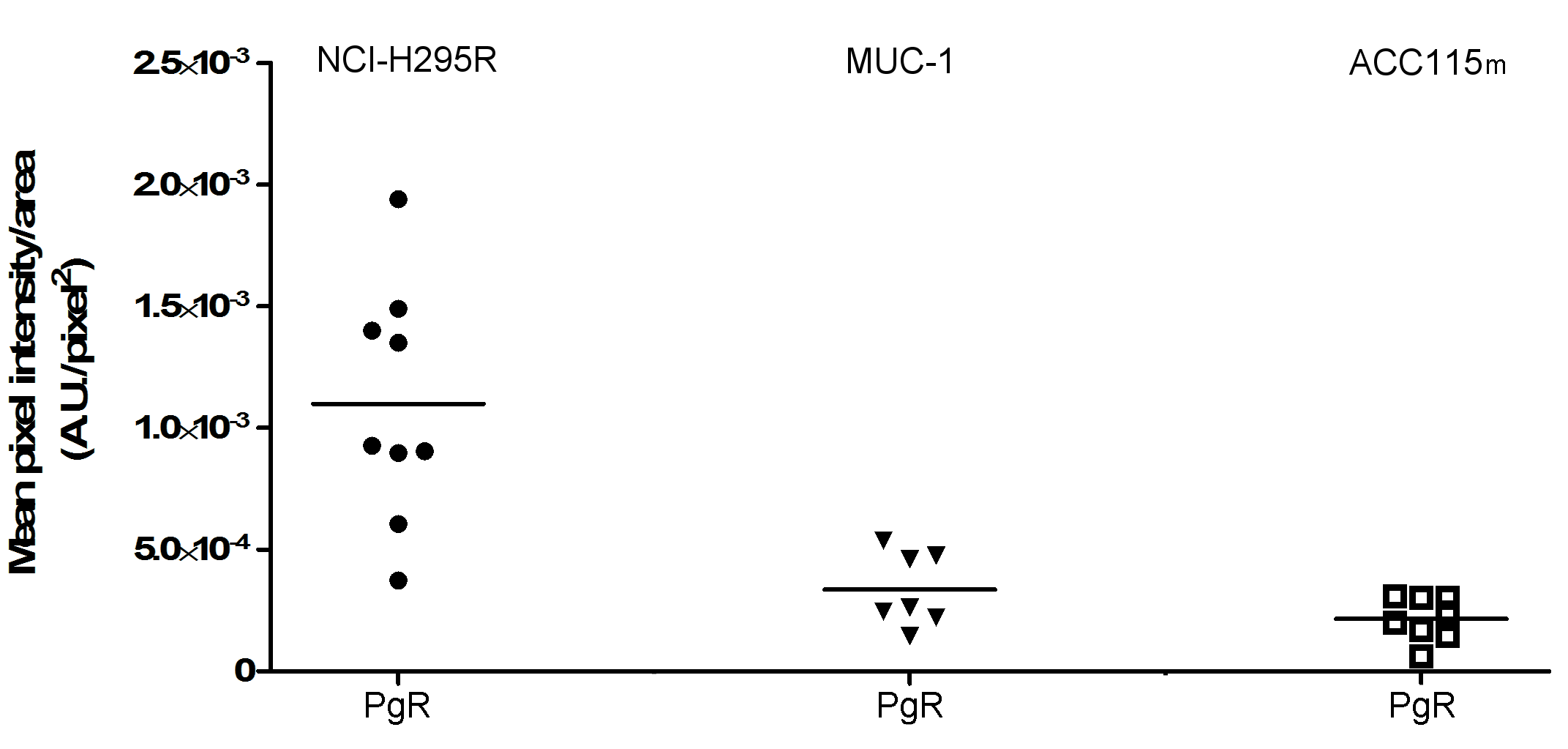
**

**Quantification of PgR basal signal in ACC cell models.** The specific mean fluorescence intensity of the pixels of acquired images was quantified using NIH ImageJ software and normalized for area in order to minimize the differences dimension among each cell lines and primary culture. Several fields, randomly chosen, were acquired, and then analyzed for each experimental condition. Quantified analysis was conducted by GraphPad Prism 5.02 software.

**Supplemental figure 5**


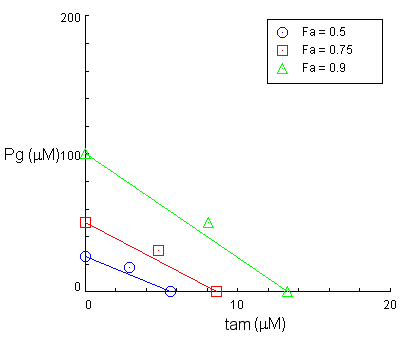


**Isobologram of tamoxifen plus Pg combination treatment in NCI-H295R cells.** Cells were treated for four days with tamoxifen plus progesterone in a constant ratio, as describe in methods. Combination data point on the diagonal line indicates additive effects, on the lower left indicates additive effects, on the upper right indicates antagonism.

**Supplemental figure 6**


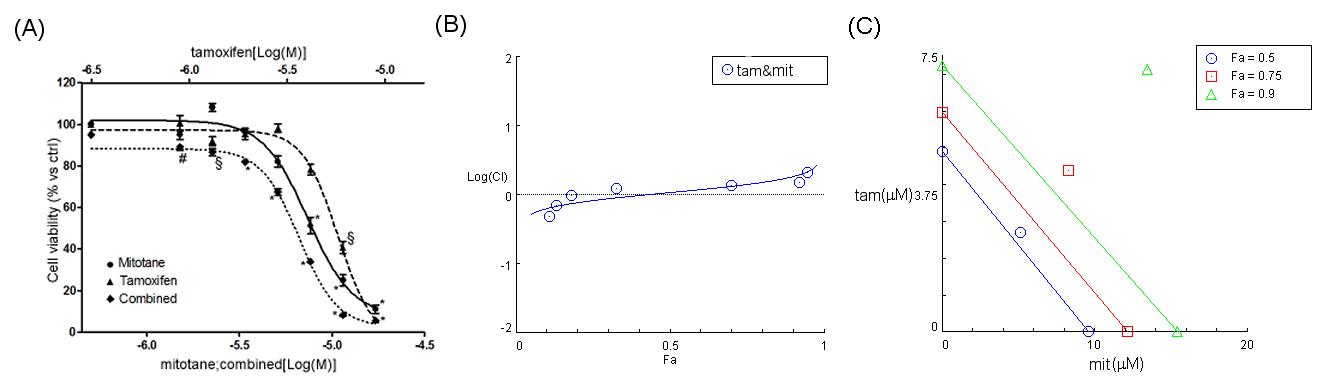


**Combined treatment of tamoxifen and mitotane in NCI-H295R cells.** (A) Cells were treated with increasing concentrations of tamoxifen (0.8 – 9.11 µM) and mitotane (1.51 – 17.21 µM) at 1:1.89 fixed molar ratio for 4 days and the cytotoxicity was assessed by MTT assay. Data are the mean ± SEM of three independent experiments performed in triplicate. **P* < 0.0001 vs ctrl; ^#^*P* < 0.001 vs ctrl; *^§^P* < 0.01. (B) The combination index was evaluated as describe in methods and the CI plot revealed an additive/synergistic cytotoxic effect at low concentrations of both drugs, for Fa ranged 0.10927 – 0.18062, while as the concentrations and the Fa values increased, the drug combination displayed an antagonist effect. The drugs concentrations with the respective combination index values are reported in Supplemental Table 3. (C) Isobologram for combination treatment: combination data point on the diagonal line indicates additive effects, on the lower left indicates additive effects, on the upper right indicates antagonism.

**3. Supplementary Tables**

**Supplemental Table 1. Clinical and immunohistochemical characteristics of ACC patients**

| **Code** | **Tumor specimen** | **Histology** | **Disease**  **stage** | **Hormone hypersecretion** |
| --- | --- | --- | --- | --- |
| **ACC03** Male 57yr | Abdominal metastasis | Mitotic index: NA,  Ki-67: NA | IV | NA |
| **ACC04** Female 62 yr | Abdominal metastasis | Mitotic index: >50/50 HPF,  Ki-67: 50% | IV | No |
| **ACC06** Male 39 yr | Abdominal relapse | Mitotic index: NA,  Ki-67: 20-40% | IV | No |
| **ACC07** Female 46 yr | Primary tumor | Mitotic index: 10/HPF,  Ki-67: 70% | IV | No |
| **ACC08** Female 39 yr | Abdominal metastasis | Mitotic index: NA,  Ki-67: NA | IV | No |
| **ACC10** Female 45 yr | Abdominal relapse | Mitotic index: 32/20 HPF,  Ki-67: >20% | IV | No |
| **ACC11** Female 70 yr | Abdominal relapse | Mitotic index: 20-30/20 HPF,  Ki-67: 15% | IV | Androgens |
| **ACC12** Female 48 yr | Thoracic metastasis | Mitotic index: Na,  Ki-67: NA | IV | NA |
| **ACC13** Male 55 yr | Primary tumor | Mitotic index:>50/10 HPF,  Ki-67: 40% | IV | No |
| **ACC14** Female 54 yr | Primary tumor | Mitotic index: >5/50 HPF,  Ki-67: <5% | II | NA |
| **ACC16** Male 54 yr | Primary tumor | Mitotic index: >50/10 HPF,  Ki-67: 50% | IV | NA |
| **ACC17** Male 47 yr | Primary tumor | Mitotic index: 2/50 HPF,  Ki-67: 25% | III | Cortisol |
| **ACC23** Female 58 yr | Primary tumor | Mitotic index: 25/10 HPF,  Ki-67: 15% | III | Cortisol |
| **ACC24** Female 44 yr | Primary tumor | Mitotic index: <1/10 HPF,  Ki-67: 3-5% | IV | Androgens |
| **ACC26** Female 30 yr | Abdominal metastasis | NA | IV | No |
| **ACC27** Female 24 yr | Lung metastasis | NA | IV | Cortisol |
| **ACC29** Female 51 yr | Primary ACC | Mitotic index: 2/50 HPF;  Ki-67: <5% | IV | Cortisol |
| **ACC30** Male 39 yr | Primary tumor | Mitotic index: 5-15/10 HPF,  Ki-67: 5-25% | IV | No |
| **ACC32** Male 66 yr | Primary ACC | Mitotic index: >5/50 HPF;  Ki-67: 20% | II | No |
| **ACC38** Female 34 yr | Primary tumor | Mitotic index: >50/10 HPF,  Ki-67: 5-60% | II | NA |
| **ACC40** Female 30 yr | Primary tumor | Mitotic index: 10-12/10 HPF,  Ki-67: 5-10% | IV | Androgens |
| **ACC48** Female 19 yr | Abdominal metastasis | Mitotic index: NA,  Ki-67: 2% | III | Cortisol |
| **ACC50** Female 28 yr | Abdominal metastasis | Mitotic index: >20/50 HPF,  Ki-67: 40% | IV | Cortisol |
| **ACC55** Male 57 yr | Peritoneal metastasis | Mitotic index: Na,  Ki-67not homogenous:  8-10%; 15-20% | IV | No |
| **ACC64** Female 60 yr | Abdominal metastasis | Mitotic index: NA,  Ki-67: 50-60% | IV | Cortisol |
| **ACC68** Female 53 yr | Abdominal metastasis | Mitotic index: NA,  Ki-67: 60% | III | Cortisol  Androgens |
| **ACC71** Female 29 yr | Abdominal metastasis | Mitotic index: NA,  Ki-67: 5% | IV | Androgens  Aldosterone |
| **ACC74** Female 78 yr | Primary tumor | Mitotic index: <1/50 HPF,  Ki-67: <1% | NA | NA |
| **ACC75** Female 55 yr | Primary tumor | Mitotic index: 15/10 HPF,  Ki-67: 25-30% | IV | No |
| **ACC79** Male 72 yr | Abdominal metastasis | Mitotic index: NA,  Ki-67: 10-15% | III | NA |
| **ACC81** Male 59 yr | Primary tumor | Mitotic index: NA,  Ki-67: 70% | IV | No |
| **ACC85** Male 45 yr | Abdominal metastesis | Mitotic index: NA,  Ki-67: 20% | IV | Estrogens |
| **ACC91** Female 62 yr | Abdominal metastasis | Mitotic index: NA,  Ki-67: 20% | IV | No |
| **ACC99** Male 82 yr | Abdominal metastasis | Mitotic index: NA,  Ki-67: 85% | IV | No |
| **ACC103** Male 41 yr | Primary tumor | Mitotic index: 3-4/10 HPF,  Ki-67: 15% | III | Cortisol |
| **ACC115** Male 57 yr | Linfonodal metastasis | Mitotic index: 58/10 HPF;  Ki-67: 22% | IV | No |

NA = Not Available

**Supplemental table 2. Sequences of gene oligonucleotide primers for qRT-PCR**

| **Gene** |  | **Oligonucleotide Sequence (5’-3’)** | **bp** |
| --- | --- | --- | --- |
| β-ACTIN | F  R | TCTTCCAGCCTTCCTTCCTG CAATGCCAGGGTACATGGTG | 146 |
| ER- α | F  R | CCACCAACCAGTGCACCATT  GGTCTTTTCGTATCCCACCTTTC | 108 |
| ER-β | F  R | AGAGTCCCTGGTGTGAAGCAAG  GACAGCGCAGAAGTGAGCATC | 143 |
| PgR | F  R | CGCGCTCTACCCTGCACTC  TGAATCCGGCCTCAGGTAGTT | 121 |
| SF-1 | F  R | CAGCCTGGATTTGAAGTTCC  TTCGATGAGCAGGTTGTTGC | 232 |

**Supplemental table 3. Combination of tamoxifen with Pg in NCI-H295R against cell viability.**

| Tamoxifen [µM] + Pg [µM] | | Fa | CI |
| --- | --- | --- | --- |
| *NCI-H295R* | | | |
| 1.2 | 7.4 | 0.14383 | 1.31207 |
| 1.8 | 11.1 | 0.24449 | 1.37847 |
| 2.7 | 16.7 | 0.44228 | 1.28165 |
| 4 | 25 | 0.69325 | 1.10010 |
| 6 | 37.5 | 0.86501 | 0.97243 |
| 9 | 56.4 | 0.86157 | 1.48011 |
| 13.5 | 84.3 | 0.97214 | 0.95459 |

Fa = Fraction affected; CI = Combination Index.

**Supplemental table 4. Combination of tamoxifen with mitotane in NCI-H295R against cell viability.**

| Tamoxifen [µM] + Mitotane [µM] | | Fa | CI |
| --- | --- | --- | --- |
| *NCI-H295R* | | | |
| 0.8 | 1.51 | 0.10927 | 0.48330 |
| 1.2 | 2.26 | 0.13303 | 0.69379 |
| 1.8 | 3.4 | 0.18062 | 0.96923 |
|  |  |  |  |
| 2.7 | 5.1 | 0.32583 | 1.24645 |
| 4.05 | 7.65 | 0.7 | 1.37401 |
| 6.075 | 11.475 | 0.91976 | 1.51086 |
| 9.11 | 17.21 | 0.94534 | 2.09133 |

Fa = Fraction affected; CI = Combination Index.
